# Neuroscience of Internet Pornography Addiction: A Review and Update

**DOI:** 10.3390/bs5030388

**Published:** 2015-09-18

**Authors:** Todd Love, Christian Laier, Matthias Brand, Linda Hatch, Raju Hajela

**Affiliations:** 1 Society for the Advancement of Sexual Health, Ardmore, PA 19003, USA; 2 Department of General Psychology: Cognition, University of Duisburg-Essen, Duisburg 47057, Germany; E-Mails: christian.laier@uni-due.de (C.L.); matthias.brand@uni-due.de (M.B.); 3 Erwin L. Hahn Institute for Magnetic Resonance Imaging, Essen 45141, Germany; 4 Private Practice, Santa Barbara, CA 93103, USA; E-Mail: linda@montecitonet.com; 5 Health Upwardly Mobile Inc., Calgary, AB T2S 0J2, Canada; E-Mail: rhajela@humassociates.net; 6 Diagnostic and Descriptive Terminology Action Group (DDTAG), American Society of Addiction Medicine (ASAM), Chevy Chase, MD 93101, USA

**Keywords:** internet pornography addiction, internet addiction, internet gaming disorder, neuroscience, neuroimaging, DSM-5, behavioral addiction, addictive behavior, cybersex, online sexual behavior

## Abstract

Many recognize that several behaviors potentially affecting the reward circuitry in human brains lead to a loss of control and other symptoms of addiction in at least some individuals. Regarding Internet addiction, neuroscientific research supports the assumption that underlying neural processes are similar to substance addiction. The American Psychiatric Association (APA) has recognized one such Internet related behavior, Internet gaming, as a potential addictive disorder warranting further study, in the 2013 revision of their Diagnostic and Statistical Manual. Other Internet related behaviors, e.g., Internet pornography use, were not covered. Within this review, we give a summary of the concepts proposed underlying addiction and give an overview about neuroscientific studies on Internet addiction and Internet gaming disorder. Moreover, we reviewed available neuroscientific literature on Internet pornography addiction and connect the results to the addiction model. The review leads to the conclusion that Internet pornography addiction fits into the addiction framework and shares similar basic mechanisms with substance addiction. Together with studies on Internet addiction and Internet Gaming Disorder we see strong evidence for considering addictive Internet behaviors as behavioral addiction. Future research needs to address whether or not there are specific differences between substance and behavioral addiction.

## 1. Introduction

A revolutionary paradigm shift is occurring in the field of addiction that has great implications for assessment and treatment. While “addiction” has historically been associated with the problematic overconsumption of drugs and/or alcohol [1], the burgeoning neuroscientific research in this field has changed our understanding over the last few decades. It is now evident that various behaviors, which are repeatedly reinforcing the reward, motivation and memory circuitry are all part of the disease of addiction [2,3,4,5,6,7,8,9,10]. Common mechanisms among addiction involving various psychoactive substances such as alcohol, opioids and cocaine; and pathological behaviors such as uncontrolled gambling, internet use, gaming, pornography and sexual acting out have also been delineated.

As a result of the growing neuroscientific evidence, the American Society of Addiction Medicine (ASAM) formally expanded their definition of addiction in 2011 to include both behaviors and substances:

Addiction is a primary, chronic disease of brain reward, motivation, memory and related circuitry. Dysfunction in these circuits leads to characteristic biological, psychological, social and spiritual manifestations. This is reflected in an individual pathologically pursuing reward and/or relief by substance use and other behaviors.

[11]

The American Psychiatric Association (APA) also acknowledged the phenomenon of behavioral addiction, as can be seen in multiple passages within the DSM-5. For example, the “Substance Related Disorders” chapter was renamed “Substance Use and Addictive Disorders”, a “Non-Substance-Related Disorders” subchapter was created, and perhaps most notably, Gambling Disorder (formerly named Pathological Gambling) was moved into this the newly formed subchapter, due to its “reflecting evidence that gambling behaviors activate reward systems similar to those activated by drugs of abuse and produce some behavioral symptoms that appear comparable to those produced by the substance use disorders” [12]. Further, a diagnosis of Internet Gaming Disorder (IGD) was placed within Section 3—Conditions for Further Study of the DSM-5. In support of this new diagnosis, the APA stated in their press release/fact sheet on IGD:

The studies suggest that when these individuals are engrossed in Internet games, certain pathways in their brains are triggered in the same direct and intense way that a drug addict’s brain is affected by a particular substance. The gaming prompts a neurological response that influences feelings of pleasure and reward, and the result, in the extreme, is manifested as addictive behavior.

[13]

This statement is supported by large amounts of neuroscientific research, as illustrated within this review. Unfortunately, the APA went on to make the following statement in the Differential Diagnosis section of IGD:

Excessive use of the Internet not involving playing of online games (e.g., excessive use of social media, such as Facebook; viewing pornography online) is not considered analogous to Internet gaming disorder, and future research on other excessive uses of the Internet would need to follow similar guidelines as suggested herein.

[12]

This decision is inconsistent with existing and emerging scientific evidence, and the conducted review aims at contributing to the ongoing discussion of Internet pornography addiction (IPA) in response to the APA’s request.

The APA has not clearly stated why the larger diagnosis, Internet Addiction (IA), was reworked into the more content specific diagnosis of IGD. This position is consistent with Davis’s [14] original concept of Specific Problematic Internet Use (SPIU), as well as Brand, Laier and Young’s [15] updated version of Specific Internet Addiction (SIA). This also matches Griffiths proposed differentiation between addictions *to* the Internet and addictions *on* the Internet [16]. An easier and perhaps more functional decision, however, would have been to maintain the proposed diagnosis of IA but simply *require* a subtype or specifier; gaming, pornography, social networking, shopping, *etc*. The exact same criteria, references, and most of the wording currently listed for IGD could have been kept, with only the word “behavior” used in lieu of the word “gaming”. Indeed, the original formal proposal for IA to be included in the DSM-5 incorporated the subtypes of instant messaging, pornography use, and video games [17], expanded later to include social networking [18]. This would have aligned the DSM-5 with what has, in fact, occurred in the field since its publication, namely, the continued scientific investigation into the broad range of potentially problematic behaviors involving Internet use. This inclusive approach has been proposed multiple times, both historically [17] and recently [19,20].

Conceptualizing IA as a generalized problem with more specific subtypes is ripe for formal reconsideration. There is a key element found throughout all internet-related experiences: The ability to maintain or heighten arousal with the click of a mouse or swipe of a finger. Attention to novelty (scanning for salient cues in the environment) furthers survival, and research shows that it activates the brain’s reward system [21]. Thus, the act of seeking (which would include surfing) triggers the reward system [22]. So do stimuli that violate expectations (positive or negative) [23], which is often found in today’s videogames and internet pornography.

Some internet activities, because of their power to deliver unending stimulation (and activation of the reward system), are thought to constitute supernormal stimuli [24], which helps to explain why users whose brains manifest addiction-related changes get caught in their pathological pursuit. Nobel prize winning scientist Nikolaas Tinbergen [25] posited the idea of “supernormal stimuli”, a phenomenon wherein artificial stimuli can be created that will override an evolutionarily developed genetic response. To illustrate this phenomenon, Tinbergen created artificial bird eggs that were larger and more colorful than actual bird eggs. Surprisingly, the mother birds chose to sit on the more vibrant artificial eggs and abandon their own naturally laid eggs. Similarly, Tinbergen created artificial butterflies with larger and more colorful wings, and male butterflies repeatedly tried to mate with these artificial butterflies in lieu of actual female butterflies. Evolutionary Psychologist Dierdre Barrett took up this concept in her recent book *Supernormal Stimuli: How Primal Urges Overran Their Evolutionary Purpose* [26]. “Animals encounter supernormal stimuli mostly when experimenters build them. We humans can produce our own.” [4] (p. 4). Barrett’s examples range from candy to pornography and highly salted or unnaturally sweetened junk food to highly engaging interactive video game playing. In short, generalized internet chronic overuse is highly stimulating. It recruits our natural reward system, but potentially activates it at higher levels than the levels of activation our ancestors typically encountered as our brains evolved, making it liable to switch into an addictive mode [27].

In the review that follows we will first provide an overview of the major theoretical understanding or models of addiction involving substances and of the neuroscientific basis on which the addictive processes work, whether there is involvement with substances or behaviors. We will then review the existing neuroscientific studies relating to the behavioral aspects of addiction generally, then the more specific problem of Gambling Disorder, and then progress to the flood of recent studies on IA, and its subtypes of gaming and pornography. The majority of studies discussed examined key aspects of addiction involving behaviors through laboratory investigation, including functional neuroimaging studies and structural and resting-state neuroimaging studies. These bear on the established science relating to addiction generally. Where relevant, we have also discussed neuropsychological studies, which suggest laboratory behavioral parallels with brain studies such as those on structural brain abnormalities thought to be the result of addiction.

We have chosen to narrow our focus primarily to the neuroscientific research findings relating to addiction involving behaviors, despite the fact that there is also a large and growing body of research relating to their clinical presentation, epidemiology, health effects, public health ramifications, *etc*. While that line of research overwhelmingly supports the prevalence and risks associated with Internet and Internet related addiction, it is outside the scope of this neuroscience focused review. Thus we believe that it makes sense to limit this review primarily to the studies that meet the most rigorous requirements, studies that address the neurobiochemical and neurophysiological processes known to underlie addiction generally.

We hope that the articles reviewed here will make it clear that the dozens of studies supporting IA (and each of its subtypes) as being neuroscientifically similar to substance addiction and will demonstrate that all of the possible Internet behaviors must be considered as potentially addictive in the same way, as variations on a theme rather than separate disorders, just as diverse forms of gambling (e.g., casinos, electronic gambling, and fixed-odds betting) may each produce recognizable signs, symptoms, and behaviors that indicate addiction. In particular we will highlight the emerging studies investigating IGD and IPA as major subtypes. In fact it is the case that most IA studies around the world have considered the various Internet behaviors in this light.

## 2. Method

To conduct the research, an extensive literature search and review was performed utilizing a variety of sources: Multiple EBSCO collections (including ERIC, LISTA, PsychARTICLES, PsychEXTRA, PsychINFO, Psychology and Behavioral Sciences and SocINDEX), Google Scholar, PubMed and multiple ProQuest collections (including Central, Dissertations and Thesis, Psychology and Social Science). A universal inclusion criterion was publication in a peer-reviewed journal. A secondary inclusion criterion was based on publication date, with differing time-delimitations set based on the specific topic/category being investigated (see details below). Continuous rechecks of the more rapidly emerging subject areas (e.g., Internet-related addictions) were performed in an effort to remain current with the expanding body of knowledge. As such, an exact number of results reviewed was impossible to calculate as the rechecks often returned results already reviewed. Some manual screen of ambiguously titled papers was required (performed by first author). Additionally, articles on treatment, etiology, psychopathology, comorbidity, or other counseling/psychological concerns regarding Internet-related addictions were eliminated, as were articles regarding Internet-related addictions as a societal or sociological issue. The reference management tool Zotero was used to build a database of all articles considered.

### 2.1. Neurobiology of Addiction

The scope of this topic was limited to the previous ten years, with primary focus given to articles published in the past five years. Older publications considered key developments within the scientific advancement of this field were also included (for example, Blum *et al*. 1990; Nestler, Barrot and Self, 2001; Robinson and Berridge, 1993; Solomon and Corbit, 1974). The following search terms and their derivatives were used in multiple combinations with database wildcards (*) as needed: Addict* (to allow for both addict, addicted, and addiction), DeltaFosB, genetic*, epigenetic*, imaging, neurobiolog* (to allow for both neurobiology and neurobiological), neuroscien* (to allow for neuroscience and neuroscientific), “reward deficiency syndrome”, and “substance* abuse*”.

### 2.2. Neurobiology of Addictive Behaviors

This scope was not time-delimited, as it is an emerging topic whose entire historical context is relevant. Analytical priority, however, was given to literature reviews, and articles published via a newest to oldest methodology. The following search terms and their derivatives were used in multiple combinations: Addict*, behavior* (to allow for both behaviors and behavioral), compulsive, imaging, non-drug, non-substance, and neurobiolog*.

### 2.3. Gambling Disorder

Gambling Disorder/Pathological Gambling has been a highly published topic for many years, and the time scope of this topic was the most limited, as it has already been accepted as an addictive behavior, and was thus restricted to neuroimaging studies or reviews published in the previous five years. Multiple combinations of the following search terms and their derivatives were used in conducting the research: Compulsive, disorder, gambl* (to allow for both gambling and gamblers), “pathological gambl*”, “problem* (to allow for both problem and problematic) gambl*”, and “neurobiolog* gambl*”.

### 2.4. Internet Addiction

As this is another emerging topic, there was no time-scope set for this topic, although priority was given to studies and reviews published in the previous five years. Special attention to nomenclature was required here, as the disorder is studied under different headings. For example, in addition to the primary term of Internet Addiction, additional terms include “Compulsive Internet Use” [28,29,30,31,32,33], Internet Addiction Disorder [34], Internet Use Disorder [35], “Pathological Internet Use” [14,36], and “Problematic Internet Use” [37,38,39,40,41,42]. As such, the following search terms and their derivatives were used in multiple combinations: Addict*, compulsive, “compulsive internet”, cyber, Internet, “Internet use”, online, “pathological internet” and “problem* Internet” (to allow for problem and problematic).

### 2.5. Internet Gaming Disorder

No time-limitation was placed on this topic, and the following search terms and their derivatives were used in multiple combinations: Game, games, gamers, gaming, “compulsive game/es/ers/ing), “online game/es/ers/ing”, and “problem* game/es/ers/ing”. All IGD references in the DSM-5 were reviewed. A less-than-exhaustive final selection approach was taken based on the fact that the APA has already approved IGD as a research-worthy diagnosis, and thus the full volume of articles in this subject area was not needed to support our premise.

### 2.6. Internet Pornography Addiction

Research into the area of addictive sexual behaviors on the Internet began with an inquiry into the various constructs surrounding compulsive sexual behavior. There was no specific time-delimitation for this search, however, as with behavioral addiction, analytical priority was placed upon literature reviews and articles published via a newest to oldest methodology. The following search terms and their derivatives were used in multiple combinations: “Compulsive sex”, cybersex, hypersexual, “hypersexual disorder”, imaging, “impulsive sex”, neurobiolog*, “out of control sex”, “problem* sex*”, sex, “sex addict*”, “sexually explicit material”, and “visual sexual stimuli”.

There was no time scope placed upon the research into the area of IPA, although a large amount of manual screening was required, as many results were articles about Internet pornography (IP) but focused on sub-topics unrelated to addictive/compulsive/problematic usage (e.g., content analysis, feminism, freedom of speech, morality concerns, societal impact, *etc*.). Additional screening was required to differentiate articles about IP (included) and non-IP (not included). Multiple combinations of the following search terms and their derivatives were used: Porn* (to allow for porn, pornographic, and pornography), addict*, compulsive, cyber, imaging, Internet, neurobiol*, online, problem*.

## 3. Literature Review

### 3.1. Neurobiology of Addiction

All drugs of abuse affect the mesolimbic dopamine (DA) pathway, which originates from the ventral tegmental area (VTA) and projects into the nucleus accumbens (NAcc). Commonly called the reward center, the NAcc is heavily connected with pleasure, reinforcement learning, reward seeking, and impulsivity. The mesolimbic dopamine pathway connects with three other key regions to form a collection of integrated circuits commonly called the reward system: The amygdala (positive and negative emotions, emotional memory), hippocampus (processing and retrieval of long term memories), and the frontal cortex (coordinates and determines behavior). Taken together, the reward system and its connecting regions modulate, among other things, pleasure, reward, memory, attention, and motivation [43].

Naturally occurring behaviors such as eating and sex, have evolved such that they activate the reward system due to the fact that they reinforce behaviors necessary for survival [20]. The past decade has yielded multiple theories of addiction, all of which involve the reward system and related brain regions and substrates [44].

#### 3.1.1. Three-Stage Model of Addiction

Nora Volkow describes addiction as a neurobiochemically based shift from impulsive action learned through positive reinforcement to compulsive actions learned through negative reinforcement [43]. This in turn is seen as leading to an addictive cycle that progressively worsens over time. Volkow, Wang, Fowler, Tomasi and Telang [43] describe three stages of the addictive cycle; (a) binge/intoxication; (b) withdrawal/negative affect; and (c) preoccupation/anticipation.

Volkow, Wang, Fowler, Tomasi and Telang [43] refer to stage one as the “Binge/Intoxication” stage. Different classes of drugs activate the reward system through different means, however, the universal result is a flood of dopamine in the NAcc (reward center). This results in acute positive reinforcement of the behavior that initiated the flood. In this impulsive stage, this positive reinforcement results in addictive related learning associations [45]. Neuroplastic changes begin to occur, however, as the continued release of dopamine in the NAcc leads to an increase in dynorphin levels. Dynorphin, in turn, decreases the dopaminergic function of the reward system, resulting in a decrease of the reward threshold and an increase in tolerance [43,45].

In stage two—“Withdrawal/Negative Affect”—the dopamine flood has run its course, and there is activation of the extended amygdala, an area associated with pain processing and fear conditioning. The resulting negative emotional state leads to activation of brain stress systems and dysregulation of anti-stress systems. This leads to a decreased sensitivity to rewards and an increase in the reward threshold, which is called tolerance. This further progresses to negative reinforcement as the individual continues to engage in the addictive behaviors to avoid the negative affect associated with withdrawal. This, in turn, encourages the reinstatement and/or reinforcement of the addictive behaviors. Here, the impulsive behavior shifts to compulsive behavior, referred to in the model as chronic taking/seeking [43,45]. A key point of this stage is that withdrawal is not about the physiological effects from a specific substance. Rather, this model measures withdrawal via a negative affect resulting from the above process. Aversive emotions such as anxiety, depression, dysphoria, and irritability are indicators of withdrawal in this model of addiction [43,45]. Researchers opposed to the idea of behaviors being addictive often overlook or misunderstand this critical distinction, confusing withdrawal with detoxification [46,47].

A second component of the reward system comes into play here; the mesocortical dopamine pathway. Like the mesolimbic DA pathway, the mesocortical DA starts in the VTA, however it terminates in the frontal cortex. Specific affected areas within the prefrontal cortex include the dorsolateral prefrontal cortex (DLPFC), responsible for key components of cognition and executive function, and the ventromedial prefrontal cortex (VMPFC) responsible for components of inhibition and emotional response. Taken together, the mesocortical dopamine pathway affects the cognitive component of reward processing [43,45].

This leads to stage three—“Preoccupation/Anticipation”—frequently referred to as craving. The neuroplastic impairments expand beyond the mesocortical dopamine pathway into other regions of the prefrontal cortex responsible for motivation, self-regulation/self-control, delayed reward discounting, and other cognitive and executive functions [43,45]. Goldstein and Volkow [48] developed the Impaired Response Inhibition and Salience Attribution (I-RISA) model to emphasize the importance of this process. The I-RISA model integrates the increased salience of learned drug-related cues (resulting from the aforementioned positive and negative reinforcement of the addictive behavior) with newly developed deficiencies in top-down inhibitory control. This leaves the individual vulnerable to reinstatement of the behavior, and two primary mechanisms have been identified; cue-induced reinstatement and stress-induced reinstatement [43,45]. Numerous neuroimaging studies substantiate this model [49,50], and these impairments are the source behind the “chronic relapsing disorder” element of the medical definition of addiction [11,51].

#### 3.1.2. Anti-Reward

George Koob proposed an expansion of the second stage of addiction. Koob [51] expands Solomon and Corbit’s [52] opponent-process model of motivation, which posits emotional experiences as opposing pairs, operating in a similar manner to the positive reinforcement transitioning to negative reinforcement shown in stages one and two of the three stage model above. In the opponent-process model of motivation, a-processes reflect positive hedonic effects and b-processes reflect negative hedonic effects. The application in addiction is that a-processes occur first and reflect tolerance. In contrast, the b-processes arise after the a-process have concluded and reflect withdrawal. Solomon and Corbit [52] used skydivers as an example of the opposite, wherein the novice skydivers experience great fear when they jump (b-process) and some relief when they land (a-process). As they repeat the behavior, the balance shifts such that experienced skydivers experience some fear when they jump but great relief when they land. This model has recently been proposed to explain the occurrence of non-suicidal self-injury (“cutting”) [53].

Koob [51] overlays a detailed biologic model onto the psychological opponent-process theory. Steps one and two of the three stage model involve “within-system changes”, marked by decreased reward system function, consisting of an increased reward threshold and a decreased natural release of dopamine to non-addictive rewards. Koob expands the model to incorporate “between-system changes”, based largely on the concept of opponent-processes. Specifically, the “Anti-Reward” theory posits that when the brain reward system is engaged, there is a parallel engagement of the brain stress systems for the purpose of limiting the reward response and the maintenance of homeostatic balance with the reward system, resulting in the activation of both the body’s stress system (the hypothalamic-pituitary-adrenal axis), and the brain’s stress system (corticotrophin-releasing factor (CRF)) system. The aforementioned elevated levels of dynorphin further elevate CRF, and the engagement of these systems brings about many of the negative affects linked to the withdrawal stage. Compounding the problem, the brains anti-stress system also becomes dysregulated, as evidenced by decreases in neuropeptide Y (a natural anxiolytic in the brain). The addicted brain enters an “allostatic” state when the reward system is unable to return to its homeostatic (normal) state. The reward system subsequently develops an altered set-point, leaving the individual vulnerable to relapse and dependence. This is what Koob calls the “dark side” of addiction [51].

#### 3.1.3. Neurobiology of Learning, Habit, and Motivation

While both the Anti-Reward and I-RISA models include learning components, other theories of addiction focus primarily on the learning aspects of addiction, and the biological underpinnings thereof. Hyman [54] refers to addiction as the “pathological usurpation of neural processes that normally serve reward-related learning” [54] (p. 565).

Everitt and Robbins [55,56] propose a model of addiction as a steady transition from voluntary actions to habitual actions to compulsive actions. Their model includes a combination of classical Pavlovian stimulus-response conditioning and instrumental learning, and they presented evidence illustrating a shift in brain activity from the ventral striatum (location of the NAcc) to the dorsal striatum (brain region established for compulsive behaviors) through the course of the development of addiction.

Robinson and Berridge [4,57] expand the learning model with the “Incentive Salience” theory of addiction. The Incentive Salience theory follows the framework of a hypersensitized mesocorticolimbic DA pathway, however, this theory focuses on the motivational attributions attached to the behavior, rather than pleasure or reward [58]. This model perhaps best follows the evolutionary function of the reward system, wherein “drugs induce a false signal of a fitness benefit, which bypasses higher-order information processing” [59]. This theory explicitly differentiates “liking” and “wanting” in that the development of addiction progresses along a path of liking (hedonic reward value) to wanting (motivational adjustment based on salience) [60,61]. The researchers thus refer to addiction as a “pathological motivation” [4] resulting in the core behavioral symptoms of addiction. These authors surmised that “bolstered by the evidence that has accumulated over recent years, we remain confident in concluding that at its heart, addiction is a disorder of aberrant incentive motivation due to drug-induced sensitization of neural systems that attribute salience to particular stimuli” [4]. Although primary focused on addiction to chemicals, these authors concluded that natural rewards are inherently connected to the dopaminergic reward system, and thus “incentive sensitization can also sometimes spill over in animals or humans to other targets, such as food, sex, gambling, *etc.*” [4].

Robinson and Berridge [61] recently updated their model to remove the necessity of the component of liking, illustrating wanting as the only component of Incentive Sensitization theory. They did so by transitioning lab rats from “revulsion” (pressing lever dispensed bitter sea salt) to “wanting”, by activating the mesocorticolimbic pathway immediately prior to the presentation of the same lever. They thus propose these results as countering the traditional Pavlovian conditioning based arguments regarding the learning component of addiction (that compulsion and cravings are based on prior learned associations), and emphasize how cravings “hijack” brain circuits of reward [61] (p. 282).

#### 3.1.4. Genetics

Genetics, as they are relevant here, can be divided into three mechanisms; Genetic heritability, addiction related genetic expression in the individual, and epigenetics intersecting the two. In regards to studies of genetic heritability, Swendsen and LeMoal [62] estimated genetic factors to contribute to approximately 40% of the disease of addiction. The authors went on to provide gender specific heritability estimates for specific substances as; 49% (m) and 64% (f) for alcohol, 44% (m) and 65% (f) for cocaine, 33% (m) and 79% (f) for marijuana, 43% (m) for opiates, and 53% (m) and 62% (f) for tobacco [62] (p. 80). Volkow and Muenke [63] report common genetic factors on both sides of dual diagnoses; for example, ADHD and substance abuse. Agrawal and colleauges [64] performed a literature review and identified addiction related genes as belonging in one of two categories; genes that potentiate metabolic changes in response to specific substances, and genes that influence reward-system behaviors (such as DRD2). These authors also found that early stages of addictive process were more tied to environmental factors, while later stages were more tied to heritability.

Blum *et al*. [65] identified the genetic connection between the A1 allele of the Dopamine D2 receptor gene (DRD2) and a susceptibility to develop alcoholism. Specifically, they held that carriers of the DRD2-A1 gene have fewer D2 receptors. A few years later, Blum, Cull, Braverman and Comings [66] proposed that individuals with this genetic predisposition are likely to have disruptions in the mesolimbic reward system, which they referred to as the “Dopamine Reward Cascade”. These interruptions result in a hypodopaminergic state that yields a predisposition to addictive, compulsive, and impulsive behaviors, as well as several personality disorders. Blum *et al*. [66] coined the term “Reward Deficiency Syndrome” (RDS) to represent the inborn chemical imbalance that presents as one or more behavioral disorders. As they continued the research, Blum and his team discovered that carriers of the DRD2-A1 gene have 30%–40% less D2 receptors, and make up about 33% of the US population [67].

#### 3.1.5. Molecular Underpinnings of Addiction

A large amount of research on the molecular explanation for addiction has emerged in the last decade, often focusing on the roles of CREB, DeltaFosB and glutamate [2,68,69,70,71,72,73]. The sum of this research indicates that the flooding of dopamine in the reward system triggers an increase in the production of cyclic AMP (cAMP), a small molecule that then signals the release of cAMP response element-binding protein (CREB). CREB is a protein that regulates the expression of specific genes. In this case, the result is the release of dynorphin, a protein that slows the release of dopamine and inhibits the VTA, thereby dampening the reward system. Researchers believe this to be the molecular basis of tolerance, as increased amounts of the drug (or behavior) are required to overcome the increased amounts of CREB. This process is also involved with dependence, as the inhibited reward system leaves the individual in a state of anhedonia when abstinent from the source of problematic dopamine release. When the addict becomes abstinent, CREB levels quickly drop, tolerance fades, and sensitization begins. At this point, DeltaFosB becomes the predominant factor.

DeltaFosB is a transcription factor that operates partially in an opposite manner to CREB, in that it suppresses dynorphin and increases sensitivity in the reward pathway. Whereas CREB results in negative reinforcement of addictive behavior, DeltaFosB promotes positive reinforcement of addictive behavior. Whereas CREB builds up quickly in response to drug use (or addictive behaviors), DeltaFosB builds up slowly. Additionally, whereas elevated CREB levels dissipate quickly, the elevated levels of DeltaFosB remain for extended periods—weeks or months. This enhances response to rewards and reward related cues, leaving the individual sensitive to addiction related cues and vulnerable to compulsive behaviors and relapse. This extended persistence and its associated implications have led to DeltaFosB’s reference as the “molecular switch for addiction” [70].

A third component is the neurotransmitter glutamate. Researchers are finding glutamate to be intimately involved with the learning component of addiction, and the increased amount of dopamine in the mesocorticolimbic pathway leads to an increased sensitivity to glutamate. In turn, the enhanced glutamate sensitivity strengthens and fuels the learning/memory pathways related to the addiction and its surrounding behaviors [74].

### 3.2. Neurobiology of Addictive Behaviors

Koob and Le Moal [5] dedicated the final section of their highly detailed review of the allostatic brain reward/anti-reward system to the topic of “Nondrug Addictions”. The authors intertwined “non-drug and drug addictions”, and concluded with the statement, “A case can be made that there is strong face validity with the addiction cycle of preoccupation/anticipation (craving), binge/intoxication, and withdrawal/negative affect stages for compulsive gambling, compulsive shopping, compulsive eating, compulsive sexual behavior, and compulsive exercise” [5] (p. 46).

In a literature review comparing addictive behaviors and SUDs, Grant, Brewer and Potenza [6] specific referenced pathological gambling, kleptomania, pyromania, compulsive buying, and compulsive sexual behavior as examples of addictive behaviors, and concluded, “Biochemical, functional neuroimaging, genetic studies, and treatment research have suggested a strong neurobiological link between behavioral addictions and substance use disorders” [6] (p. 92). Grant, Potenza, Weinstein and Gorelick [7] found addictive behaviors and SUD’s to overlap in multiple areas, including comorbidity, course (chronic relapse), genetic contribution, neurobiology (brain glutamatergic, opioidergic, serotonergic, dopamine mesolimbic systems), phenomenology (craving, intoxication, withdrawal), tolerance, and treatment response.

In his detailed article, “Natural rewards, neuroplasticity, and non-drug addictions”, Olsen [8] declared, “there is a glut of evidence that natural rewards are capable of inducing plasticity in addiction-related circuitry” [8] (p. 14). Olsen cited fMRI studies showing gambling, shopping, sex (orgasm), video games, and the sight of appetizing food to activate the mesocorticolimbic system and extended amygdala in the same manner as do drugs of abuse. Olsen concluded that “Extensive data suggests that eating, shopping, gambling, playing video games, and spending time on the internet are behaviors that can develop into compulsive behaviors that are continued despite devastating consequences” [8] (p. 14).

In their review of the genetic heritability of behavioral addiction, Lobo and Kennedy [75] reported pathological gamblers to be three times more likely to have a parent who is a pathological gambler, and twelve times more likely to have grandparent. Blum *et al*. [67] found children of alcoholics to be 50%–60% more likely to become alcoholics, a statistic that exactly matches Leeman and Potenza’s [10] heritability rate for pathological gamblers.

Blum has consistently included addictive behaviors in his constellation of domains impacted by RDS. In an early paper on the reward cascade, Blum *et al*. [76] stated, “Therefore lack of D2 receptors causes individuals to have a high risk for multiple addictive, impulsive and compulsive behavioral propensities, including alcoholism, cocaine, heroin, marijuana and nicotine use, glucose bingeing, pathological gambling, sex addiction...”. The following list represents specific behavioral problems currently tied to RDS (please note here that we use the original terms, although we would not categorize Internet Gaming or Aberrant Sexual Behavior under the term Compulsive Behaviors):

- Addictive Behaviors: Severe Alcoholism, Polysubstance Abuse, Smoking and Over Eating—Obesity
- Impulsive Behaviors: Attention-Deficit Disorder Hyperactivity, Tics and Tourette Syndrome and Autism (including Asperger Syndrome)
- Compulsive Behaviors: Aberrant Sexual Behavior, Internet Gaming and Obsessive Texting, Pathological Gambling and Workaholism and Shopaholisnm
- Personality Disorders: Conduct Disorder, Antisocial Personality, Aggressive Behavior, Pathological Cruelty and Violence [67].

According to Smith [77], brain science studies such as these and others led to ASAM’s inclusion of behaviors into its formal definition of addiction. In addition to the previously mentioned “Short Definition of Addiction”, ASAM published a “Long Definition of Addiction”, wherein they provides specific examples of addictive behaviors in the first paragraph:

Addiction also affects neurotransmission and interactions between cortical and hippocampal circuits and brain reward structures, such that the memory of previous exposures to rewards (such as food, sex, alcohol and other drugs) leads to a biological and behavioral response to external cues, in turn triggering craving and/or engagement in addictive behaviors.

[11]

In further support of the concept of addiction involving behaviors, ASAM uses the phrase “Addictive behaviors” 13 times in their Long Definition of Addiction, and expounds upon the phrase in Explanatory footnote 3:

In this document, the term “addictive behaviors” refers to behaviors that are commonly rewarding and are a feature in many cases of addiction. Exposure to these behaviors, just as occurs with exposure to rewarding drugs, is facilitative of the addiction process rather than causative of addiction. The state of brain anatomy and physiology is the underlying variable that is more directly causative of addiction. Thus, in this document, the term “addictive behaviors” does not refer to dysfunctional or socially disapproved behaviors, which can appear in many cases of addiction. Behaviors, such as dishonesty, violation of one’s values or the values of others, criminal acts etc. can be a component of addiction; these are best viewed as complications that result from rather than contribute to addiction.

[11]

Research on the neurobiology of “behavioral addictions” has continued since the time of the new ASAM definition. For example, in their literature review of the epidemiology, neurobiology, and treatment options of “behavioral addictions” [9], Karim and Chaudhri indicated an increased legitimacy of the disorders, which they also refer to as impulsive-compulsive behaviors, and process addictions. These authors specifically referenced “gambling, eating, sex, shopping, use of the Internet or videogames or even exercising, working or falling in love” [9] (p. 5) as examples of behavioral addictions.

Leeman and Potenza [10] conducted a thorough literature review of the neurobiological studies on addictive behaviors, “A Targeted Review of the Neurobiology and Genetics of Behavioural Addictions: An Emerging Area of Research”. This article contains 197 references, and breaks the findings down into three categories: Brain function and neuroimaging results, neurotransmitter systems, and genetics. The authors summarized each category into its own full-page table, outlining six “behavioral addictions”: Gambling, Internet, gaming, shopping, kleptomania, and sex. The left column of the table included a summary of the existing research on the specific behavioral addiction, and the right column contrasted them with corresponding findings for substance abuse. The authors concluded that there is limited but emerging data connecting different behavioral addictions with existing research on substance abuse.

Fineberg *et al*. [78] published an extensive review, “New developments in human neurocognition: Clinical, genetic, and brain imaging correlates of impulsivity and compulsivity”. In their review, these top authors de facto acknowledge the concept of addictive behaviors, including them in their endeavor to “understand(ing) of the pathophysiology of impulsive, compulsive, and addictive disorders and indicate new directions for research” [78] (p. 2). These authors used Gambling Disorder as the reference model for behavioral addictions, although they next acknowledged binge-eating disorder as showing a common neuropathophysiology with substance addictions. Included in their findings, these authors report,

As in alcohol dependence, an inverse relationship between ventral striatal activation during reward anticipation and self-reported impulsivity was observed in both the pathological-gambling and alcohol-dependent groups suggesting that this feature of blunted ventral striatal activation across behavioral- and substance-addiction groups relates similarly to impulsivity.

[78] (p. 15)

The concept of food as addictive has been particularly studied in recent years, including heavy research into the neurobiological components of binge eating and obesity [79,80,81,82,83,84,85,86,87,88,89,90].

#### 3.2.1. Gambling Disorder

In addition to the aforementioned research into the neurobiology of both substance use disorders (SUDs) and addictive behaviors, there is a substantial body of research specifically into the neurobiology of Gambling Disorder (GD) (known as Pathological Gambling (PG)) prior to the DSM-5). Indeed, as mentioned in the Fineberg *et al*. [78] study, many studies on addictive behaviors use GD as the prototype.

Other studies directly compared and contrasted the neurobiology of GD with the neurobiology of SUDs. For example, Potenza [91,92] published two literature reviews specific to the neurobiology of GD. In his first literature review, investigating commonalities between GD and substance abuse, Potenza [92] found similarities to extend to clinical, genetic, epidemiological, phenomenological, and other biological domains, and raised the question as to whether GD would be more appropriately categorized as a “behavioral addiction”. These findings are reinforced in his second study, in which he found multiple brain regions (ventral striatum, ventromedial prefrontal cortex, insula, among others), and neurotransmitter systems (norepinephrine, serotonin, dopamine, opioid, and glutamate) to be altered in disordered gamblers [91].

Building upon such research, Leeman and Potenza [10] published a review on the “Similarities and differences between pathological gambling and substance use disorders”. The authors illustrated multiple similarities between GD and SUDs in regards to brain function (frontal cortices, striatum, and insula) and neurotransmitter system research findings (dopamine, serotonin, opioids, glutamate, and norepinephrine). Similarly, el-Guebaly and colleagues published a review investigating the appropriateness of fit of GD as an impulse control disorder or as an additive disorder [93]. Based on findings of applicable neurotransmitters, neurocircuitry, and genetics as well as response to pharmacotherapies, these authors found that GD and SUDs had more in common than between GD and impulse control disorders. Similarly, Brevers and Noël [94] published a literature review where they found GD to fit within the I-RISA, Anti-Reward, Incentive Salience/Sensitization, and habit models of addiction. As a final example, Gyollai *et al*. [95] published a literature review on the genetics of GD and conclude by validating its inclusion in the RDS constellation of behaviors.

Based on this and multitudes of other research, the APA reclassified Pathological Gambling from being an Impulse Control Disorder to a “Non-Substance Related Disorder” in the DSM-5. This recognition of GD as a non-substance-related disorder (*i.e.*, Behavioral Addiction) in the DSM-5 represents the breaking down of the long-standing assumption that the scientific studies of addiction and the concept of addiction in general must be limited to the pathological use of psychoactive substances.

Since that time, neuroimaging studies and reviews continue to emerge. For example, Singer *et al*. [96] reviewed the studies relating to the neurobiological underpinnings of GD based on the idea that the recent re-classification of GD as a Behavioral Addiction in the DSM-5 suggested that “similar cognitive and motivational phenotypes may underlie both gambling and substance use disorders” [96] (p. 1). In particular they described a number of studies which lend support to the idea that exposure to reward unpredictability can cause aberrant responses in the dopamine systems, which in turn mediates incentive salience to reward-related cues. The reviewers also touched upon studies suggesting that cortisol plays a role in modulating incentive motivation in the ventral striatum, *i.e*., that cortisol levels in gambling addicts positively correlate with ventral striatal responses to monetary cues.

Finally, a recent review by Romanczuk-Seiferth *et al*. [97] started from the premise that there was already an increasing body of literature showing neurobiological similarities between GD and SUDs, and that this is further supported by the fact that specific treatments for SUDs are also effective in treating gambling addicts. They examined the recent neuropsychological, neurophysiological, and neuroimaging studies of GD based on the three main clusters of diagnostic criteria: Loss of control, craving/withdrawal and neglect of other areas in life”. They concluded that grouping these symptom clusters in this way provided “a useful framework for systematic comparisons of new evidence in GD and SUDs in the future” [97] (p. 95).

#### 3.2.2. Internet Addiction

Researchers have been studying IA for nearly two decades. Kimberly Young presented the first empirical research on IA at the American Psychological Association’s annual conference in 1996, and there have been hundreds of studies and reviews on the topic conducted since that time. There have been at least 20 literature reviews published in the last five years on the broad topic of IA, and/or its specific subtypes [15,36,47,98,99,100,101,102,103,104,105,106,107,108,109,110,111,112,113]. Among these reviews, at least 10 have reviewed, in part or in whole, the research on the neurobiological findings regarding IA [15,104,105,111,114,115,116,117,118,119].

In their literature review on the neurobiology of “Internet and Gaming Addiction”, published prior to the release of the DSM-5, Kuss and Griffiths [105] noted;

Internet addiction comprises a heterogeneous spectrum of Internet activities with a potential illness value, such as gaming, shopping, gambling, or social networking. Gaming represents a part of the postulated construct of Internet addiction, and gaming addiction appears to be the most widely studied specific form of Internet addiction to date.

[105] (p. 348)

Nonetheless, there is an unfortunately pervasive conflation of the conceptual problem of “Internet addiction” and “Internet gaming disorder”. For example, APA openly confused the concept of IA with its subtype of IGD in the DSM-5 when they stated “Internet gaming disorder (also commonly referred to as Internet use disorder, Internet addiction, or gaming addiction) has merit as an independent disorder” ([12], p. 796). The APA furthered this conflation via the 14 references for IGD they provided in the DSM-5 to support the diagnosis. Thirteen of these references were to peer-reviewed journals, and one is a reference to a pop-culture magazine article (“Wired”) about IA in China. Among the peer-reviewed articles, only three articles were actually specifically focused on Internet Gaming [120,121,122]. Of the 10 remaining articles, four studies referred to gaming as one of three subtypes of IA [34,116,123,124], one referenced gaming as one of ten subtypes [125], three made use of the terms “game” and “gaming” interwoven with other Internet related terms such as “gambling” and “pornography” [126,127,128], and two referred to “Internet use” generally with no subtypes [129,130].

Despite the APA’s reformulation, a number of researchers, including prolific neurobiology researcher Guangheng Dong, have continued to refer to IGD as a subtype of IA [131,132,133,134,135]. In a more recent review, released after the publication of the DSM-5, Brand, Young and Laier [15] stated:

The APA has now focused on Internet gaming. We argue, however, that also other applications can be used addictively...Therefore, we summarize results of previous studies on Internet addiction in a broader way, although a great proportion of studies published so far concentrated on Internet gaming.

[15] (p. 2)

Similarly, for the purposes of this review, any study that conceptualizes IGD as a subtype of IA is classified as an IA study for the purposes of this review, although many use gaming as the prototypical example. For example, Weinstein and Lejoyeux [116] reviewed articles exclusively on “Internet addiction” and “problematic Internet use” published in Medline and PubMed between 2000–2009. While this study was not specific to neurobiology, these authors briefly reported on findings in this area, concluding:

The results demonstrated that the neural substrates of cue-induced gaming urge/craving in online gaming addiction was similar to that of the cue-induced craving in substance dependence. Thus, the results suggested that the gaming urge/craving in online gaming addiction and craving in substance dependence might share the same neurobiological mechanism.

[116] (p. 279)

Kuss and Griffiths [105] published a literature review on the neurobiology of “Internet and Gaming Addiction”, in which they cite a mix of studies that are either specific to subjects addicted to internet gaming or subjects that are Internet addicted without any specific subtype identifier. Similarly Weinstein and Lejoyeux’s review [115] “New developments on the neurobiological and pharmaco-genetic mechanisms underlying Internet and videogame addiction” contains the phrase “Internet and videogame addiction” consistently throughout their paper, although the scope of their review is specific to gaming. Regardless of nomenclature inconsistencies, it is critical to note that much of the results of both reviews are directly in line with many of the aforementioned neurobiology of addiction findings [4,43,44,51,55,56,57,61]. As part of these findings, the mesocorticolimbic reward system was found to be impacted in the same manner as with substance abuse, as was the cue-induced craving phenomenon.

Researchers from the National Institute of Psychiatry in Mexico also conducted a review on the topic of IA. These researchers investigated the classification, comorbidity, diagnosis, electrophysiology, epidemiology, molecular genetics, neuroimaging, and treatment (pharmacological and non-pharmacological) of the disorder. Based on their findings, the researchers concluded that “considerable clinical and neurobiological research has been done on the subject...with research pouring in data from different parts of the world” [111] (pp. 1, 7). Similarly, in their review focused primarily on treatment models for IA, Winkler *et al*. [118] also reported a “substantial overlap with the symptoms commonly associated with behavioral addictions and neurological similarities with other addictions [118] (p. 326)”.

One recent review focused on the role of prefrontal control functions in IA and summarized neuropsychological and neuroimaging studies on this topic [15]. The authors assumed that IA can be differentiated into generalized IA and several specific IAs, e.g., IGD or IPA. In line with the aforementioned addiction models [4,43,44,51,55,56,57,61], and particularly based on recent results from neuroimaging studies in Internet addicted individuals, the authors concluded that IA seems to be related with structural and, more prominent, functional brain changes in cortical (e.g., prefrontal cortex and limbic structures) and subcortical (e.g., parts of the basal ganglia) brain areas. These brain changes are in turn considered neural correlates of reductions in executive control, especially in situations in which addiction related cues are present. Brand *et al*. introduced a cognitive-behavioral model of generalized and specific IA emphasizing positive and negative reinforcement due to Internet use, which lead to cue-reactivity and craving reactions. The authors posited that the processes of cue-reactivity and craving might accelerate the problems in executive control functions [15].

Meng and colleagues [114] conducted the first literature review/meta-analysis combination of fMRI studies of IGD. These authors started with 61 articles, which they consolidated to 10 voxel-wise whole-brain analysis studies. The authors find a key commonality of prefrontal lobe dysfunction, and thus conclude, “Considering the overlapped role of prefrontal lobe in the reward and self-regulatory system, our results provided supportive evidence for the reclassification of IGD as a behavioral addiction” [114] (p. 799).

In another recent literature review on the neurobiology of IA, Zhu, Zhang and Tian [119] specifically reviewed molecular mechanisms through neuroimaging studies utilizing functional magnetic resonance imaging (fMRI), positron emission tomography (PET) and single photon emission computed tomography (SPECT). These authors found that IA is associated with dysfunction in the brain dopaminergic systems just like addiction involving substances; and MRI studies have shown structural changes in the brain in IA subjects, with the impaired cognition and behavioral control found in IGD adolescents specifically, being associated with structural brain changes in the pre-frontal cortex and insula that are characteristic of addiction.

A burgeoning number of studies on the genetics of IA are emerging. For example, Montag *et al*. [136] claimed they may have found a molecular indicator of IA via the gene coding for the nicotinic acetylcholine receptor subunit alpha 4 (CHRNA4). These researchers found a significant increase in a specific polymorphism on the CHRNA4 gene in the Internet addicted subjects. Moreover, Lee *et al*. [137] found internet addicted subjects to have higher SS-5HTTLPR frequencies. Additionally, Han *et al*. [138] found Internet addicted subjects to have significantly more prevalent Taq1A1 alleles, low activity COMT alleles and higher reward-dependence scores relative to controls.

The most recent IA reviews focused only on neuroimaging studies while omitting relevant EEG studies. Our search has additionally identified 15 IA EEG studies, four specific to IGD. In the study of addictive behaviors, both resting state EEG and event related potentials may be employed. Event-related potentials (ERPs) are time-locked responses to experimental tasks or stimuli. For example, Yu, Zhao, Li, Wang and Zhou [139] tested subjects using auditory oddball tasks and found reduced P300 amplitudes and increased P300 latencies in IA subjects compared to healthy controls. Decreased P300 have been reported in other substance abusers [140], and suggest poorer memory and attention allocation. The authors also reported a weakening of gamma oscillation intensity, which has been demonstrated is related to reduced dopamine levels. Similarly, Duven, Müller, Beutel and Wölfling [141] conducted a study involving a game in which participants received rewards. The IGD group had significantly lower P300 amplitudes during the finding of rewards, leading the authors to conclude that the blunted P300 reflected deficits in IGD subjects reward system, a finding in line with substance addictions. Ge *et al*. [142] employed auditory oddball task and also found significantly increased P300 latencies. These authors found these P300 latency increases to return to normal levels after subjects completed a three-month CBT program. A second longitudinal study reported abstinence along with treatment improved short-term memory and normalization of P300 amplitudes and latencies [143]. These last two studies suggest that cognitive changes may a consequence of IA.

Zhou, Yuan, Yao, Li and Cheng [144] tested subjects using visual Go/No-Go tasks and reported greater impulsivity and lower N2 amplitudes in IA subjects compared to healthy controls. Lower N2 amplitudes in neuropsychological tests parallel findings in alcohol use disorder [145]. These researchers stated in their conclusion, “The results of this study clearly show that individuals with PIU were more impulsive than controls and shared neuropsychological and ERPs characteristics of some disorders, such as pathological gambling, drug addiction, ADHD or alcohol abuse...” [145] (p. 233). Similarly, Dong, Zhou and Zhao [146] reported that IA subjects relative to controls exhibited lower NoGo N2 amplitude and longer P300 latency. Additionally, Yang, Yang, Zhao, Yin, Liu and An [147] found that IA subjects, similar to substance abusers, engaged more executive functions in NoGo tasks. A Go/No-Go paradigm involving “excessive gamers” produced comparable results [148]. Finally, Yu, Zhao, Wang, Li and Wang [149] employed a keystroke mismatch task to asses N400 differences between excessive Internet users and controls. The N400 amplitude was lower in excessive Internet users, indicating potential difficulty retrieving conceptual knowledge. Similar findings have been reported for alcohol abusers and heavy cannabis users [140].

Zhou, Li and Zhu [150] used a modified Erikson flanker task, and reported decreased event-related negativity (ERN’s) in the Internet addicted subjects compared to controls. ERN’s are a subset of ERP’s and illustrate brain error when subjects attempt to control attention and impulsivity—the lower the ERN’s, the greater chance that the brain will not auto-correct faulty cognitions. The authors cited studies illustrating low ERN’s in ADHD and substance abuse, illustrating how patients have difficulty suppressing the urge to accept short-term rewards despite negative long-term consequences. Attributing the low ERN’s to deficits in executive functioning, these researchers concluded, “The results of this study clearly demonstrate individuals with Internet addiction were more impulsive than controls and shared neuropsychological and ERN characteristics of some disorders, such as pathological gambling, substance abuse...” [150] (p. 5). Yau, Potenza, Mayes and Crowley [151] employed a Balloon Analogue Risk Task (BART), and reported lower feedback-related negativity (FRN) and P300 amplitudes in “at risk problematic Internet users” compared to controls. According to these authors, less sensitivity to feedback during risk-taking may contribute to continued use despite negative consequences. Dong, Zhou and Zhao [152] tested subjects using a color-word Stroop task, and reported lower medial frontal negativity (MFN) in IA subjects compared to controls. Along with more response errors, these authors reported that this finding suggests reduced executive function, a shared feature of addictions.

A single ERP study compared cue-reactivity in excessive computer gamers and casual computer gamers. In line with substance abuse studies, Thalemann, Wölfling and Grüsser [153] found significantly higher cue-evoked ERP’s in excessive pathological players compared with casual players. Finally, two resting state EEG studies have been published. These studies reported IA subjects had lower absolute power on the delta and beta bands compared to controls. Both studies suggest these differences may be neurobiological markers for IA [154,155]. Taken together, the EEG studies provide additional evidence that those suffering from IA have much in common with those suffering from substance addictions as compared with controls.

#### 3.2.3. Internet Gaming Disorder

IA was formally proposed for inclusion in the DSM-5 two times, once with gaming as a subtype, and once with no subtypes [17,34]. IGD however, was never formally proposed for inclusion in the DSM-5, so it did not go through the formal commenting procedure. Nonetheless, in the final hour, the APA granted IGD access into Section 3—Conditions for Further Study, while IA was dismissed. There is a copious body of research on the topic of “Internet Addiction”, and it can be difficult to untangle whether studies are actually specific to IGD, or cover IA in general with gaming as a subtype. It is understandable that gaming subjects are the most often studied subtype, as much of the leading neuroscientific research into the phenomenon of IA comes from China and South Korea, countries in which IP is outlawed, and therefore research on IPA is generally lacking [156].

This review follows the original proposals, considering gaming as a subtype of IA. As this paper is primarily focused on another subtype of IA, IPA, limited attention is given to IGD as an independent subtype or disorder. As such, the reporting of neuroscience studies on both IA and IGD is combined. Despite claims of limited research on the topic [12,16,46,47,157,158,159], a yearly breakdown of primary brain studies (excluding reviews) on IA and its subtype IGD makes it evident that brain studies in support of IA in this field are mounting rapidly:

- Prior to 2009—6 studies,
- 2009—4 studies,
- 2010—8 studies,
- 2011—9 studies,
- 2012—14 studies,
- 2013—19 studies,
- 2014—23 studies, and
- 2015 (through June)—16 studies.

Categorized by technology, these brain studies comprise 44 fMRI studies [103,132,134,135,160,161,162,163,164,165,166,167,168,169,170,171,172,173,174,175,176,177,178,179,180,181,182,183,184,185,186,187,188,189,190,191,192,193,194,195,196,197,198,199], 23 structural MRI studies [124,128,131,133,200,201,202,203,204,205,206,207,208,209,210,211,212,213,214,215,216,217,218], 6 nuclear imaging (PET/SPECT) studies [117,129,219,220,221,222], 15 EEG studies [42,139,141,143,144,146,148,149,150,152,153,154,155,223,224], and 7 physiological studies [121,138,225,226,227,228,229].

This extensive neuroscientific evidence provides compelling support for the acknowledgment of internet-related addictions as valid disorders. Further, research continues to emerge on another proposed subtype of social networking/facebook addiction, however these are generally not neuroscience studies and thus not within the scope of this paper for further review [100,104,171,230,231,232,233,234,235,236,237,238,239,240,241].

#### 3.2.4. Compulsive Sexual Behavior

Childress *et al*. [242] conducted a study in which they took fMRI scans of cocaine addicted patients presented with rapid (33 millisecond), preconscious visual cues (drug-related images). The same subjects were later shown preconscious sexually related visual cues (erotic images). The researchers found activation of the same limbic system/reward circuitry in subjects shown sexual cues as when shown drug-related cues. In their literature review of the neuroimaging studies of the human sexual response cycle, Georgiadis and Kringelbach [243] concluded, “it is clear that the networks involved in human sexual behavior are remarkably similar to the networks involved in processing other rewards” [243] (p. 74).

Frascella, Potenza, Brown and Childress [244] conducted a literature review contrasting three specific behaviors with alcoholism, pathological gambling, obesity, and the mechanics of sexuality. The authors broadened the scope of the Childress *et al*. [242] study, and concluded functional brain imaging studies of sex, romantic love and attachment provide ample evidence for an extended but identifiable system central to natural, non-drug reward processes and survival functions...The overlap of classic reward brain areas involved in sexual arousal, love and attachment is complete (VTA, NAcc, amygdala, ventral pallidum, orbitofrontal cortex). Speculation is justified that associates survival-level natural rewards with substance addictions, expanding the brain systems to be addressed in therapy, and increasing our understanding of the necessary tenacity of the behaviors [242] (p. 15).

As stated previously, the RDS model includes problematic sexual behaviors in a list of RDS-related problems [245,246,247,248].

The term “Reward Deficiency Syndrome” was first coined...in 1995, and is now defined by the Microsoft Dictionary as “A brain reward genetic dissatisfaction or impairment that results in aberrant pleasure seeking behavior that includes drugs, excessive food, sex, gaming/gambling and other behaviors”.

[249] (p. 2)

Perhaps the largest volume of studies indicating a neurobiological basis for compulsive sexual behavior as akin to the addiction model involve the transcription factor DeltaFosB. It has been well established that drugs of abuse elevate levels of the transcription factor DeltaFosB in the reward system, resulting in enhanced response to rewards and reward related cues, increased sensitivity to addiction related cues, and heightened vulnerability to compulsive behaviors and relapse [2,73,250,251,252]. Note that this line of research must utilize non-human mammals, such as mice, rats, and hamsters, as a required part of the study requires euthanizing the subjects in order to access and measure intracranial DeltaFosB. For example, researchers have genetically modified mice to overproduce DeltaFosB in the reward system at similar levels to those of drug addicted mice. When presented with cocaine for the first time, these mice showed increased sensitivity to the drug and respond and behave in manners similar to those of rats who had become addicted through chronic use [253]. Multiple tests using Syrian hamsters treated to overproduce DeltaFosB have focused on the effects of sexual behavior, and found a similarly enhanced sensitivity to sexual activity [254,255]. Wallace *et al*. [256] naturally induced this sensitivity in laboratory rats via “chronic sexual behavior”. These authors found repeated sexual experience significantly increased DeltaFosB levels in the NAcc compared with controls, although the rates of increase were lesser than with drugs of abuse. Pitchers *et al*. [257] similarly illustrated the production of high levels of DeltaFosB in the NAcc, further finding this elevation to be critically involved in the reinforcing effects of sexual reward. Investigating the combination of natural and drug rewards, Pitchers *et al*. found mice to have increased sensitivity to amphetamines after repeated sexual experiences [258]. These authors concluded, “Sexual experience induces functional and morphological alterations in the mesolimbic system similar to repeated exposure to psychostimulants” [258] (p. 1). Pitchers *et al*. [2] confirmed these findings, illustrating that natural rewards (sexual behavior) and drugs of abuse (amphetamines) act on the same reward system pathways, further supporting the argument for behavioral addictions, including IPA.

#### 3.2.5. Internet Pornography

In his highly regarded book on neuroplasticity, *The Brain That Changes Itself* [259] Norman Doidge summarized the research on addiction and the reward system, and stated that the continued release of dopamine into the reward system when an individual compulsively and chronically watches Internet pornography stimulates neuroplastic changes that reinforce the experience. Doidge went on to explain how these neuroplastic changes build brain maps for sexual excitement. He introduced an additional component of tolerance, in that previously established brain maps for “natural” sexuality cannot compare to the newly developed and continuously reinforced maps generated by continued compulsive watching of Internet pornography, and thus the addicted individual progresses to more explicit and graphic Internet pornography in order to maintain the higher level of excitement.

Neurosurgeons Hilton and Watts [260] published a commentary in the Journal Surgical Neurology International which they titled “Pornography addiction: A neuroscience perspective”. The authors provided a short literature review renewing the argument that all manifestations of addiction operate via the same underlying mechanisms. The authors included many of the previously mentioned studies; the role of DeltaFosB in natural addictions, neuroanatomical changes caused by excessive behaviors, changes in dopamine receptor density, and the influence of excessive behaviors on the reward system. In their response to a rebuttal to their paper, Hilton and Watts elaborated on the importance of taking a broader view of existing research, concluding, “Our premise is that selective atrophy of cortical areas associated with reward pathways may be viewed in a neuromodulatory light, given current research confirming neuroplasticity in overindulgence in natural rewards, specifically sexuality” [261] (p. 6). Hilton published a second and similar literature review [24], again emphasizing the critical role of DeltaFosB research as informing the study of not only sexuality in general but the more specific scope of internet pornography consumption.

The first fMRI study which explicitly focused on IPA was published in 2014, when the first in a series of Cambridge University studies found the same brain activity as seen in drug addicts and alcoholics [262]. In this arguably landmark study an experiment was conducted designed to measure the subjective experience of cue-reactivity, as well as the neurobiological markers and correlates, if any, found in subjects with compulsive sexual behavior (CSB). Note that this study included two primary lines of investigation. First, the study investigated the “liking vs. wanting” distinction for CSB and non-CSB subjects. The subjects were shown the videos both inside and outside of the fMRI scanner. Each time, subjects were asked to rate their subjective experiences via two specific measures: “How much did this increase your sexual desire?” and “How much did you like this video?” [262] (p. 3). This line of study yielded two distinct results: (1) Compared to the healthy control subjects, the CSB subjects reported higher desire ratings to the sexually explicit videos, but not to the erotic clips; (2) Compared to the healthy controls, the CSB subjects reported higher liking rating to the erotic clips, but not to the explicit cues. These results indicated a dissociation between liking and wanting by CSB-subjects when watching sexually explicit videos. These results replicated the results of well-established studies on the incentive-salience theory of addiction, wherein addicts report higher levels of wanting but not of liking their salient rewards.

The second primary area of investigation contained within this study regards neuroimaging results of compulsive sexual behaviors (CSB), internet pornography in particular. Prior studies have indicated common brain regions activated during craving states and drug-cue-reactivity for alcohol, cocaine, and nicotine; among others, the amygdala, dACC, and ventral striatum [263]. While the researchers in the present study found these same regions to become activated within both CSB and non-CSB subjects when shown sexually explicit materials, the researchers found elevated activation in the CSB subjects. Based on these results, Voon *et al*. [262] concluded:

The current and extant findings suggest that a common network exists for sexual-cue reactivity and drug-cue reactivity in groups with CSB and drug addictions, respectively. These findings suggest overlaps in networks underlying disorders of pathological consumption of drugs and natural rewards”.

[262] (p. 9)

Incidentally, these researchers also reported that 60% of subjects (average age: 25 years) had difficulty achieving erections/arousal with real partners, yet could achieve erections with internet pornography. Note that this finding is in line with the actual results of a recent study purporting to find otherwise [264].

Kühn and Gallinat [263] conducted an MRI study with sixty-four healthy (non-CSB) male subjects and correlated hours of online viewing of explicit material per week and years of use with dorsal striatal structure and connectivity. Three main results were reported. First, longer duration and more hours per week of use correlated with lower grey matter volume in the right caudate. While the caudate serves multiple complex functions, volume changes in the striatum are associated with several addictions, while the direction of change is not consistent. Second, more years and more hours per week of use correlated with lower left putaminal activity in response to brief, still sexual images. fMRI studies confirmed that the putamen is activated during sexual arousal [265,266]. The authors suggested this lower volume may reflect tolerance brought about by desensitization: “This is in line with the hypothesis that intense exposure to pornographic stimuli results in a downregulation of the natural neural response to sexual stimuli” [236] (p. E6). Given the stronger response to 9-second explicit video clips in Voon *et al*. [262], it may be that brief (530 milliseconds) exposures to still images do not act as cues for today’s internet porn video viewers, and are instead a good way to measure decreasing sexual response. Alternatively, the non-addicts here examined may be responding differently than addicts would have. Finally, subjects who consumed more pornographic material were found to have less connectivity between the right caudate and left dorsolateral prefrontal cortex (DLPFC). While the DLPFC is concerned with executive functions, it is also associated with cue reactivity to drugs and internet gaming. Disruptions in this circuit are implicated in drug and behavioral addictions. Specifically, poor functional connectivity between the DLPFC and caudate (as found in the current study) is implicated in heroin addiction [267].

Multiple presentations indicating potential upcoming papers on the neurobiology of IPA were delivered at the 2015 2nd International Conference on Behavioral Addictions in Budapest, Hungary. Note that these are all conference proceedings and have not yet been published in peer reviewed journals. They do provide further proof, however, of the fact that there is a rapidly growing body of research. For example, Gola, Wordecha, Sescousse, Kossowski and Marchewka [268] presented on their fMRI study of persons with internet pornography focused CSB. These researchers followed a study model [269], in which researchers found increased sensitivity in response to addictive cues (measured by shorter reaction times) and blunted response in the ventral striatum when shown non-addictive cues. In their study, Gola *et al*. found partially similar results; CSB subjects showed significantly increased sensitivity to addictive cues (erotica) compared to controls, however they did not find a blunted response to non-addictive cues. In a similar fMRI study, Brand, Grabenhorst, Snagowski, Laier and Maderwald [270] found heterosexual males to have increased ventral striatal activity in response to preferred pornographic images. Further, the increase in activity correlated with the degree of subjective complaints due to their Internet Pornography addiction. Wehrum-Osinsky, Klucken and Stark [271] reported on a potentially similar fMRI study they conducted with 20 subjects reporting excessive internet pornography consumption and 20 control subjects. Although specific details of their study were not included in their published abstract, these authors reported finding of “altered neural processing of sexual cues in the patient as compared to the control group” [271] (p. 42).

Although more neuropsychological than neurobiological, multiple studies have been conducted investigating the impacts of internet pornography viewing on cognitive operations. This line of inquiry is relevant to the present paper in that the neurobiological mechanisms underlying neuropsychological operations have been well established. For example, Fineberg *et al*. [272] published a narrative review wherein they explored the interrelationships between multiple findings in neuroscience. In their work, these authors provided a table wherein they mapped neurocognitive domains (different forms of impulsivity and compulsivity) to neuroanatomical and neurochemical findings. Using GD as the model, these authors tied neural structures such as the orbitofrontal cortex (OFC) and subcortical connections with neurotransmitters such as serotonin and serotonin/dopamine (respectively), as determined by tasks measuring cognitive operations such as decision-making and response time. Similarly, in their aforementioned review, Fineberg *et al*. [78] reported that their findings “resonate with those from neurocognitive assessments of people with gambling and alcohol-use problems in which both groups demonstrated greater impulsivity, but the alcohol-dependent group additionally showed impairments on executive functioning thought to involve greater involvement of the DLPFC” [78] (p. 15). As such, we believe that reporting the following neuropsychological studies exploring the interference of processing sexual cues and sexual arousal with executive functions has direct applicability to this review of brain science studies focused on the problem of IPA.

Several theories and experimental paradigms have been developed to describe and investigate executive functions [273]. Generally, executive functioning describes a complex interplay between several cognitive domains in order to facilitate goal-directed behaviors, e.g., focusing attention, inhibiting (irrelevant) information, switching between (relevant) information, planning, monitoring, and coding information in working memory [274,275] which can be affected and interfered by emotional processes [273]. Regarding the neural correlates of executive functions it was shown that they generally were located in the prefrontal cortex, but vary between the single facets of executive functions [276,277,278]. Neuropsychological and neuroimaging studies on substance addictions showed that the prefrontal cortex and executive functions get impaired following substance use [46,279]. This was taken into account to explain repeated drug administration and the preference for short-term reinforcement due to the drug despite severe negative consequences following drug use [280].

Within the development of addictive sexual behaviors on the Internet it was assumed that anticipating and receiving gratification plays an important role [281], since sexual arousal is highly reinforcing [241,279]. Experimentally, it was shown that sexual arousal reactions to Internet pornographic cues were related to symptom severity of IPA in heterosexual males and females as well as in homosexual males [282,283,284,285] and that problematic IP users reacted with increased subjective craving compared to healthy cybersex users when being confronted with Internet pornographic material [286]. It has been further shown that positive implicit associations as measured by an Implicit Association Task modified with pornographic pictures [287] and moreover, approach and avoidance tendencies [288] are linked to symptoms of IPA. Based upon these observations, the model of specific internet addiction proposed by Brand *et al*. [15] has recently been specified for cybersex use (including IP) [289].

Reid, Karim, McCrory and Carpenter [290] found greater self-reported executive dysfunction in a sample of hypersexual patients, another study found no general impairments of executive functions observed using neuropsychological tests [291]. However, several studies reported an interference of the processing of sexual cues and sexual arousal with executive functions. Deficits in visual processing caused by bound attention due to erotic stimuli was shown in studies using a choice reaction time task [292], rapid target perception [293], and a dot detection task [294,295,296]. An interference with inhibition ability was demonstrated in a study using Go/No-go tasks with neutral and sexual images and showed that individuals with high sexual excitability and high impulsivity showed worse task performance [297].

In line with the above, Laier, Pawlikowski and Brand [298] used an Iowa Gambling Task modified with pornographic pictures and found that the sexual arousal in a decision making situation can interfere with feedback processing and advantageous decision making. Similarly, sexual arousal induced by sexual images impaired working memory performance in a pictorial 4-back paradigm [299] as well as switching and monitoring performance in an executive multitasking paradigm [300]. The findings of an attentional bias towards sexually explicit cues was replicated and shown to be enhanced in a sample of sexually compulsive individuals [301]. This is in line with the theoretical suggestion that executive functions should be affected in situations in which individuals are confronted with addiction-related cues eliciting craving reactions [15]. One study used EEG while participants performed a Tower of Hanoi and the Wisconsin Card Sorting Test and viewed neutral and erotic videos [302]. In the results, no difference in the task performance was observed when comparing video conditions, but differential prefrontal coupling was observed during the two tasks in the erotic video condition. The authors explain that sexual arousal interfered with cognitive functioning but that task performance was not decreased because of functional adaptations during the task performance, which in turn could be interfered with in craving situations experienced in addiction.

An EEG study on those complaining of problems regulating their viewing of internet pornography has reported the neural reactivity to sexual stimuli [303]. The study was designed to examine the relationship between ERP amplitudes when viewing emotional and sexual images and questionnaire measures of hypersexuality and sexual desire. The authors concluded that the absence of correlations between scores on hypersexuality questionnaires and mean P300 amplitudes when viewing sexual images “fail to provide support for models of pathological hypersexuality” [303] (p. 10). However, the lack of correlations may be better explained by arguable flaws in the methodology. For example, this study used a heterogeneous subject pool (males and females, including 7 non-heterosexuals). Cue-reactivity studies comparing the brain response of addicts to healthy controls require homogenous subjects (same sex, similar ages) to have valid results. Specific to porn addiction studies, it’s well established that males and females differ appreciably in brain and autonomic responses to the identical visual sexual stimuli [304,305,306]. Additionally, two of the screening questionnaires have not been validated for addicted IP users, and the subjects were not screened for other manifestations of addiction or mood disorders.

Moreover, the conclusion listed in the abstract, “Implications for understanding hypersexuality as high desire, rather than disordered, are discussed” [303] (p. 1) seems out of place considering the study’s finding that P300 amplitude was negatively correlated with desire for sex with a partner. As explained in Hilton (2014), this finding “directly contradicts the interpretation of P300 as high desire” [307]. The Hilton analysis further suggests that the absence of a control group and the inability of EEG technology to discriminate between “high sexual desire” and “sexual compulsion” render the Steele *et al*. findings uninterpretable [307].

Finally, a significant finding of the paper (higher P300 amplitude to sexual images, relative to neutral pictures) is given minimal attention in the discussion section. This is unexpected, as a common finding with substance and internet addicts is an increased P300 amplitude relative to neutral stimuli when exposed to visual cues associated with their addiction [308]. In fact, Voon, *et al*. [262] devoted a section of their discussion analyzing this prior study’s P300 findings. Voon *et al*. provided the explanation of importance of P300 not provided in the Steele paper, particularly in regards to established addiction models, concluding,

Thus, both dACC activity in the present CSB study and P300 activity reported in a previous CSB study[303] may reflect similar underlying processes of attentional capture. Similarly, both studies show a correlation between these measures with enhanced desire. Here we suggest that dACC activity correlates with desire, which may reflect an index of craving, but does not correlate with liking suggestive of on an incentive-salience model of addictions.

[262] (p. 7)

So while these authors [303] claimed that their study refuted the application of the addiction model to CSB, Voon *et al*. posited that these authors actually provided evidence supporting said model.

Another EEG study involving three of the same authors was recently published [309]. Unfortunately, this new study suffered from many of the same methodological issues as the prior one [303]. For example, it used a heterogeneous subject pool, the researchers employed screening questionnaires that have not been validated for pathological internet pornography users, and the subjects were not screened for other manifestations of addiction or mood disorders.

In the new study, Prause *et al*. compared EEG activity of frequent viewers of Internet pornography with that of controls as they viewed both sexual and neutral images [309]. As expected, the LPP amplitude relative to neutral pictures increased for both groups, although the amplitude increase was smaller for the IPA subjects. Expecting a greater amplitude for frequent viewers of Internet pornography, the authors stated, “This pattern appears different from substance addiction models”.

While greater ERP amplitudes in response to addiction cues relative to neutral pictures is seen in substance addiction studies, the current finding is not unexpected, and aligns with the findings of Kühn and Gallinat [263], who found more use correlated with less brain activation in response to sexual images. In the discussion section, the authors cited Kühn and Gallinat and offered habituation as a valid explanation for the lower LPP pattern. A further explanation offered by Kühn and Gallinat, however, is that intense stimulation may have resulted in neuroplastic changes. Specifically, higher pornography use correlated with lower grey matter volume in the dorsal striatum, a region associated sexual arousal and motivation [265].

It’s important to note that the findings of Prause *et al*. were in the opposite direction of what they expected [309]. One might expect frequent viewers of Internet pornography and controls to have similar LPP amplitudes in response to brief exposure to sexual images if pathological consumption of Internet pornography had no effect. Instead, the unexpected finding of Prause *et al*. [309] suggests that frequent viewers of Internet pornography experience habituation to still images. One might logically parallel this to tolerance. In today’s world of high-speed Internet access, it is very likely that frequent consumers of Internet pornography users view sexual films and videos as opposed to still clips. Sexual films produce more physiological and subjective arousal than sexual images [310] and viewing sexual films results in less interest and sexual responsiveness to sexual images [311]. Taken together, the Prause *et al*., and Kühn and Gallinat studies lead to the reasonable conclusion that frequent viewers of internet pornography require greater visual stimulation to evoke brain responses comparable to healthy controls or moderate porn users.

In addition, the statement of Prause *et al*. [309] that, “These are the first functional physiological data of persons reporting VSS regulation problems” is problematic because it overlooks research published earlier [262,263]. Moreover, it is critical to note that one of the major challenges in assessing brain responses to cues in Internet pornography addicts is that viewing sexual stimuli *is* the addictive behavior. In contrast, cue-reactivity studies on cocaine addicts utilize pictures related to cocaine use (white lines on a mirror), rather than having subjects actually ingest cocaine. Since the viewing of sexual images and videos is the addictive behavior, future brain activation studies on Internet pornography users must take caution in both experimental design and interpretation of results. For example, in contrast to the one-second exposure to still images used by Prause *et al*. [309], Voon *et al*. chose explicit 9-second video clips in their cue reactivity paradigm to more closely match Internet porn stimuli [262]. Unlike the one-second exposure to still images (Prause *et al*. [309]), exposure to 9-second video clips evoked greater brain activation in heavy viewers of internet pornography than did one-second exposure to still images. It is further concerning that the authors referenced the Kühn and Gallinat study, released at the same time as the Voon study [262], yet they did not acknowledge the Voon *et al*. study anywhere in their paper despite its critical relevance. 

## 4. Conclusions

This review investigated the current body of scientific knowledge regarding neural processes of addiction in relation to both broad areas of psychoactive substances and behaviors such as gambling, sex and internet use, as well as the available research supporting specific behavioral aspects and their subtypes. Most of the studies used neuroimaging measures, EEGs, or physiological measurements, although some studies used neuropsychological measures. The common thread was that they all used neural data to tie addiction involving behaviors Internet-related manifestation of addiction (and the subtypes) in particular, to the well-established neuroscience on “substance abuse”. The net result of this inquiry yielded a very large number of neuroscience based studies that support the application of the addiction model to addictive Internet-related behaviors.

ASAM clearly stated that all manifestations of addiction are about common effects on the brain, not the differences in substances or contents or behaviors. Thus, based on this and the findings reviewed within this paper, it is difficult to justify the APA’s explicit disavowal of other compulsive internet behaviors (“Excessive use of the Internet not involving playing of online games (e.g., excessive use of social media, such as Facebook; viewing pornography online)) is not considered analogous to Internet gaming disorder...” [12] (p. 797). By this logic, viewing IP excessively and playing internet games excessively are substantively different, despite substantial overlap in activation of the reward system of the brain, and despite the potential for the exhibition of similar psychosocial behaviors and psychosocial consequences. This is, “biologically and behaviorally inconsistent” [24] (p. 5).

The misunderstanding of addiction neuroscience can be further seen in the DSM-5’s Diagnostic Features section for IGD:

The essential feature of Internet gaming disorder is persistent and recurrent participation in computer gaming, typically group games, for many hours. These games involve competition between groups of players...participating in complex structured activities that include a significant aspect of social interactions during play. Team aspects appear to be a key motivation.

[12] (p. 797)

Based on this logic, abusing substances in a bar or at a party can constitute substance abuse, but abusing substances while alone does not. To make an internet-related analogy, this logic dictates that someone playing World of Warcraft excessively is addicted, but someone playing Candy Crush excessively is not. This review presents strong neuroscientific evidence for viewing internet-related behaviors, including IP use, as potentially addictive, which should be taken into consideration when discussing the classification of IPA.
